# Assessing integrative prevention at work: A scoping review

**DOI:** 10.1177/10519815251383525

**Published:** 2025-11-04

**Authors:** Andrée-Anne Drolet, Alexandra Lecours, Lily Bellehumeur-Béchamp

**Affiliations:** 1 Department of Occupational Therapy, 14847 Université du Québec à Trois-Rivières, Québec, Canada; 2 Center for Interdisciplinary Research in Rehabilitation and Social Integration, Québec, Canada

**Keywords:** occupational health, health promotion, occupational injury prevention, disability management, workplace, scoping review

## Abstract

**Background:**

Integrative prevention at work is a promising avenue to better prevent occupational injuries and manage prolonged incapacity in a changing world of work. Integrative prevention at work can be operationalized using its five defining attributes: (1) holistic vision of health, (2) common understanding of the purpose of integrative prevention, (3) communication among stakeholders, (4) collaboration among stakeholders, and (5) coordination of preventive action. An assessment tool for these characteristics would be a valuable resource for organizations seeking to improve their approach to prevention. Namely, it would allow organizations to assess the presence of integrative prevention at work in their environment and enhance their ability to implement it.

**Objective:**

This study aimed to describe the evaluation tools assessing attributes of integrative prevention at work.

**Methods:**

This scoping review followed a five-step process: 1) identifying the research question, 2) identifying relevant documents, 3) selecting documents, 4) extracting the data, and 5) examining, synthesizing, and reporting the results.

**Results:**

Twelve evaluation tools were identified assessing one or more attributes of integrative prevention at work. Descriptive elements are provided for each tool (e.g., its purpose, the attribute(s) it assesses, and its metrological properties). Our study suggests that communication among stakeholders and collaboration among stakeholders are the attributes that are the most assessed by the evaluation tools.

**Conclusions:**

This study provides the first comprehensive and detailed overview of the extant tools currently being used to assess the attributes of integrative prevention at work. None can assess all five attributes on a unified scale.

## Introduction

The contemporary world of work faces continuously evolving challenges that complicate the prevention of occupational injuries and management of incapacity at work. Multifaceted and rapidly evolving societal challenges, such as labor shortages, viruses that can lead to global pandemics, the acceleration of teleworking, and the use of artificial intelligence, add complexity to the prevention of occupational risks and their consequences on workers’ health.^1–4^ Even if some of these challenges have been present for the last decade, better solutions are needed to promote organizational and worker adaptability, ultimately ensuring the health and well-being of the workforce.^5^ Traditionally, the continuum of prevention for occupational injuries and prolonged incapacity at work is divided into different levels, such as primary prevention (i.e., actions aiming to prevent any occupational injury to occur),^6^ secondary prevention (i.e., actions aiming to halt or delay the progression of an occupational injury),^6^ and tertiary prevention (i.e., actions aiming to prevent chronicity or relapse caused by an occupational injury).^6^ Some authors^7,8^ have also included health promotion (i.e., actions aiming to empower people's control over their health and well-being) as a part of this continuum. Authors argue that this traditional approach to prevention (i.e., managing each level independently) adds unnecessary complexity to prevention efforts^9,10^ and is no longer an effective solution to the challenges of today's workplaces.^1,11–13^ These levels of prevention involve different stakeholders that may come from three main work systems: the work environment (e.g., workers, leaders and managers, union representatives, human resources professionals), the health system (e.g., rehabilitation and nursing professionals, physicians, ergonomists), and the insurance industry (e.g., private or public insurers, inspectors).^9,14–16^ Differences in the work systems and disciplines from which stakeholders come mean that they are often physically isolated from one another.^17,18^ Stakeholders have varied organizational missions (i.e., priorities of their employing organizations) as well as diverse roles in prevention efforts (e.g., supportive vs. enforcement roles), and they often act on a single level of prevention (e.g., ergonomists acting in primary prevention through workspace adaptations, occupational therapists acting in tertiary prevention through rehabilitation).^6,9,19^ These issues call for a more coordinated and comprehensive preventive approach.^6,10^ To address these issues, this article presents a scoping review identifying operational tools for organizations seeking to implement a new approach to prevention: integrative prevention at work.

**Figure 1.**
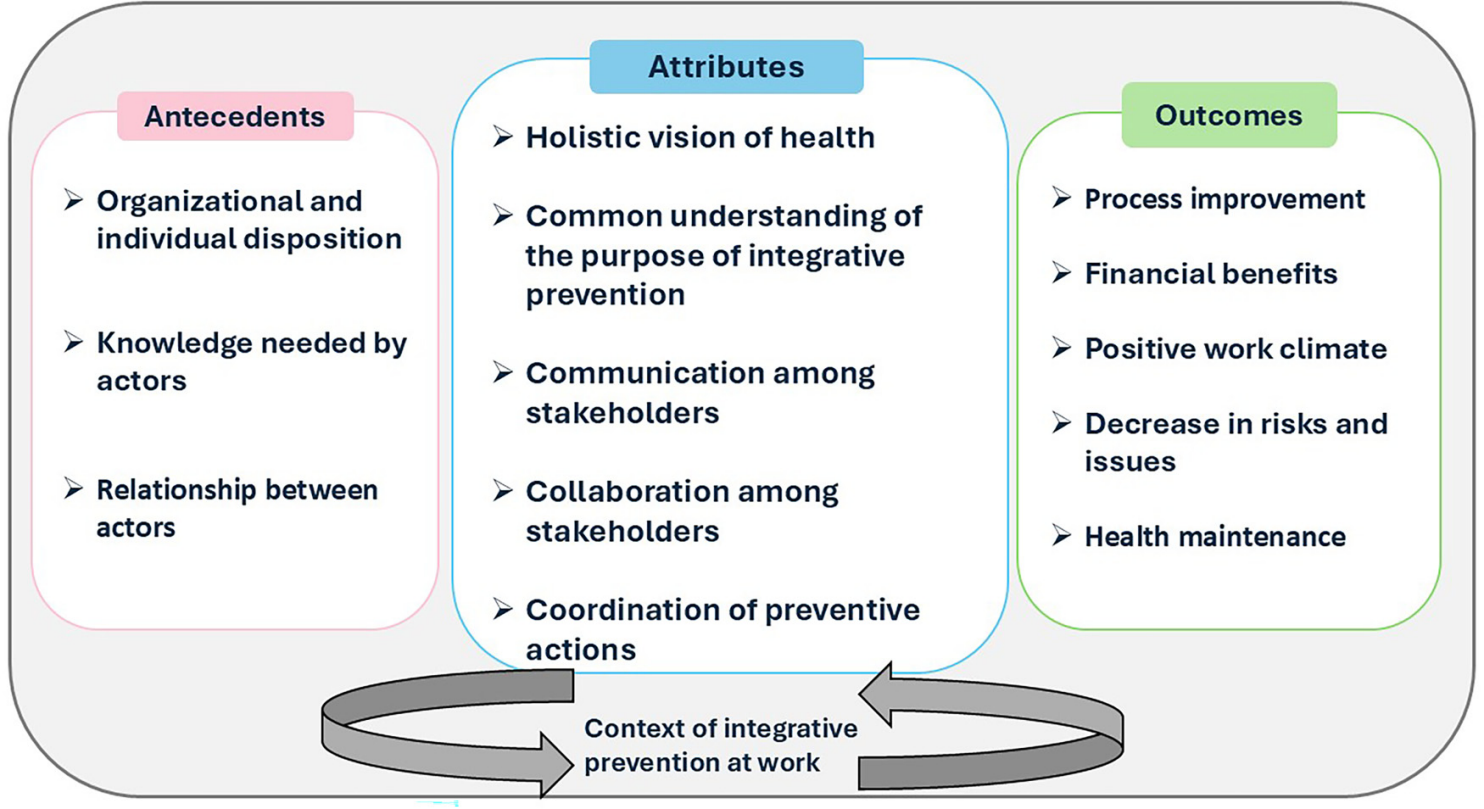
Conceptualization of integrative prevention at work, adapted from.^21^

Integrative prevention at work is an emerging concept that proposes abolishing boundaries between traditional levels of prevention (i.e., primary, secondary, tertiary) and across stakeholders involved (e.g., stakeholders from the work environment, health system, or insurance industry) to promote a more comprehensive approach characterized by interdisciplinarity.^6,10^ This concept is a promising solution proposed in the literature to encompass limitations linked to traditional means of prevention.^10,20,21^ To provide a definition of integrative prevention at work that encompasses all the essential characteristics of this concept, our research team conducted a literature review and a qualitative study.^22^ First, a meta-narrative review including 20 manuscripts identified the attributes (i.e., the manifestation of the approach in reality), antecedents (i.e., variables that precede the approach), and outcomes (i.e., variables that result from or flow from the approach) that define integrative prevention at work.^20^ This initial definition was then enriched through a qualitative study that collected perspectives of stakeholders involved in the continuum of occupational injuries and prolonged work incapacity. This process led to the following conceptualization of integrative prevention at work (see Figure 1).^21^

This conceptualization proposes that five attributes define integrative prevention at work. First, the ***holistic vision of health*** means that integrative prevention at work aims to prevent all facets of workers’ health. This means that stakeholders must address problems regarding mental health, physical health, and well-being simultaneously. This also means that beyond preventing occupational risks, workplaces and their stakeholders must foster and promote good health habits and good preventive behaviours for everyone. Second, having a ***common understanding of the purpose of integrative prevention*** means that all stakeholders must have a shared goal regarding prevention efforts. They must also agree on actions that will achieve that goal and work toward it together. Third, integrative prevention at work is also characterized by the ***communication among stakeholders***, which means that stakeholders must promote quality exchanges, and information must circulate effectively among everyone. In addition, any issue that could negatively impact workers’ health must be pointed out; it should not be silenced. This leads to the fourth attribute, ***collaboration among stakeholders***, which means that stakeholders must know their own roles and responsibilities, as well as those of others, in order to be able to engage fairly and continually. This also means that decision making must be shared regardless of the hierarchical level of the stakeholders involved, so that true collaboration is achieved. Collaboration is needed within an organization as well as with external stakeholders. Finally, ***coordination of preventive actions*** represents one of the main ideas of integrative prevention, which is to coordinate and act simultaneously on the levels of the continuum of prevention of occupational injuries and prolonged incapacity (i.e., primary, secondary, tertiary prevention, and health promotion). Furthermore, it also implies that a wide variety of actions are well integrated and that their impacts on other actions are considered. This conceptualization complements existing literature on the initial definitions of integrative prevention at work (e.g.,^6,10^), which primarily emphasize the integration of the three traditional levels of prevention, as well as recent work on the integration of health promotion and protection (e.g.,^23,24^). The attributes offer a holistic conceptualization that unifies current knowledge on health promotion and the three levels of prevention, emphasizing the importance of integrating these elements. As such, they contribute to a more comprehensive understanding of prevention, which may enhance the potential benefits for workers.^20^

As shown in Figure 1, it must be noted that integrative prevention at work is situated in a particular context, has prerequisites (i.e., antecedents) to be actualized, and promises to generate several resulting benefits (i.e., outcomes).^20,21^ These considerations justify the need to operationalize integrative prevention at work in organizations. Although a clear conceptualization of integrative prevention at work exists, organizations continue to face challenges in translating this concept into concrete action. This gap hinders the implementation of more effective prevention strategies. In our opinion, there is a need for operational tools to bridge the divide between concept and practice. Such tools could guide the transformation of current prevention practices.^20,21^

An evaluation tool that assesses the characteristics of integrative prevention at work would enhance the ability of organizations to implement this innovative preventive approach. The core attributes of a concept^25^ should be the characteristics evaluated in the first steps of the evaluation of the degree of implementation of an emerging concept.^25^ Having a tool that assesses the degree of implementation of an integrative prevention approach would be a first key step towards its actualization in the workplace.^26,27^ Many authors have emphasized the need for a unified assessment tool that could guide organizations in the implementation of integrative prevention at work.^21,26–29^ Such a tool could be used from the outset of the implementation process to establish the organization's baseline level of integrative prevention practices. This baseline could be used throughout the process as a reference point, allowing organizations to track their progress in implementation.^26^ The tool could serve to guide stakeholders by providing them with a clear understanding of the key characteristics of the approach and how to best implement them within their specific context.^19,26^ Moreover, the interpretation of the assessment tool's results regarding the approach's characteristics could also help organizations set their next objectives,^26,28^ identify organizational changes to be made,^23,26^ or even serve as a benchmark for their performance implementing the approach's characteristics.^27,30^ Beyond its potential benefits for the actualization of integrative prevention at work, having an evaluation tool for such an approach is linked to positive effects on stakeholders’ communication, sense of belonging, and engagement with one another,^31^ which are crucial components of this approach.^20^ In brief, a unified evaluation tool of attributes of integrative prevention at work would be a valuable resource for organizations seeking to implement it. However, to our knowledge, no study has yet offered an overview of existing tools assessing these attributes. We believe that identifying these tools and compiling them into a single register is the next logical step to support future developments in this emerging field and to facilitate the operationalization of this concept in organizational settings. ***Therefore, to fill that gap, the aim of this study was to describe the evaluation tools assessing attributes of integrative prevention at work****.*

## Methods

### Design

A scoping review design was selected to structure this study, which reviews a comprehensive range of literature on our topic of interest.^32–35^ Scoping reviews provide a clear and rigorous overview of extant literature and can help organize findings.^32,36,37^ In addition, scoping reviews are particularly useful for bringing together information from several disciplines,^32^ which is valuable given the interdisciplinary nature of integrative prevention at work. The selection of this design is further guided by its flexibility, which allows the inclusion of scientific literature regardless of study design, as well as gray literature (e.g., theses, reports, government texts).^32^ This is an asset for meeting the research objective, given that integrative prevention at work is an emerging approach that is still sparsely documented in the scientific literature.^20^ To increase the scientific rigor of this investigation, the PRISMA-ScR (Preferred Reporting Items for Systematic Reviews and Meta-Analyses Extension for Scoping Reviews) checklist was used to report the information.^38^

### Procedure and analysis

In this study, five systematic steps guided the research process, according to the guidelines of Arksey & O’Malley^32^ and the newest recommendations of Colquhoun et al.,^33^ Levac et al.,^34^ and Daudt et al.^35^ for conducing scoping reviews

#### Step 1: identifying the research question

The research question for this study was: “**What are the evaluation tools to assess the attributes of integrative prevention at work?”** This research question is wide in order to include as many relevant manuscripts as possible without losing focus on the objective.^32–35^ The goal is to include literature that presents evaluation tools that assess one or more attributes of integrative prevention at work. The inclusion criteria for the manuscripts were as follows: Each manuscript must (1) address the theme of work, (2) address the evaluation or measurement of integrative prevention, or at least one of its attributes, and (3) address the prevention of occupational injuries and/or management of prolonged incapacity. For feasibility reasons, we retained documents only in French or English. We imposed no publication deadline. The inclusion criteria for the eligibility of manuscripts were pretested on 20 abstracts by two members of the research team.^37^

#### Step 2: identifying relevant manuscripts

A literature search strategy was developed in collaboration with a consulting librarian specialized in the field of work to determine the relevant keyword combinations (e.g., “Integrat* prevention” AND workplace* AND “assessment tool*”) for conducting the scoping review. Databases in the fields of psychology (e.g., PsycINFO), rehabilitation (e.g., CINAHL), human resource management, and industrial relations (e.g., ABI/INFORM Global, Business Source Complete) were targeted to explore the subject, because these are the fields from which the conceptualization of this approach arises.^20,21^ Next, in alignment with the chosen study design,^32^ a Google search using the same keywords as those used in scientific databases was carried out to access manuscripts from gray literature. The references of the first five pages were explored considering the relevance ranking of the results generated during a web search.^39^ Finally, the reference lists of all selected manuscripts were manually reviewed to identify additional interesting manuscripts. To ensure literature saturation, this step was repeated until no new manuscript was identified.^32^

#### Step 3: study selection

**Figure 2.**
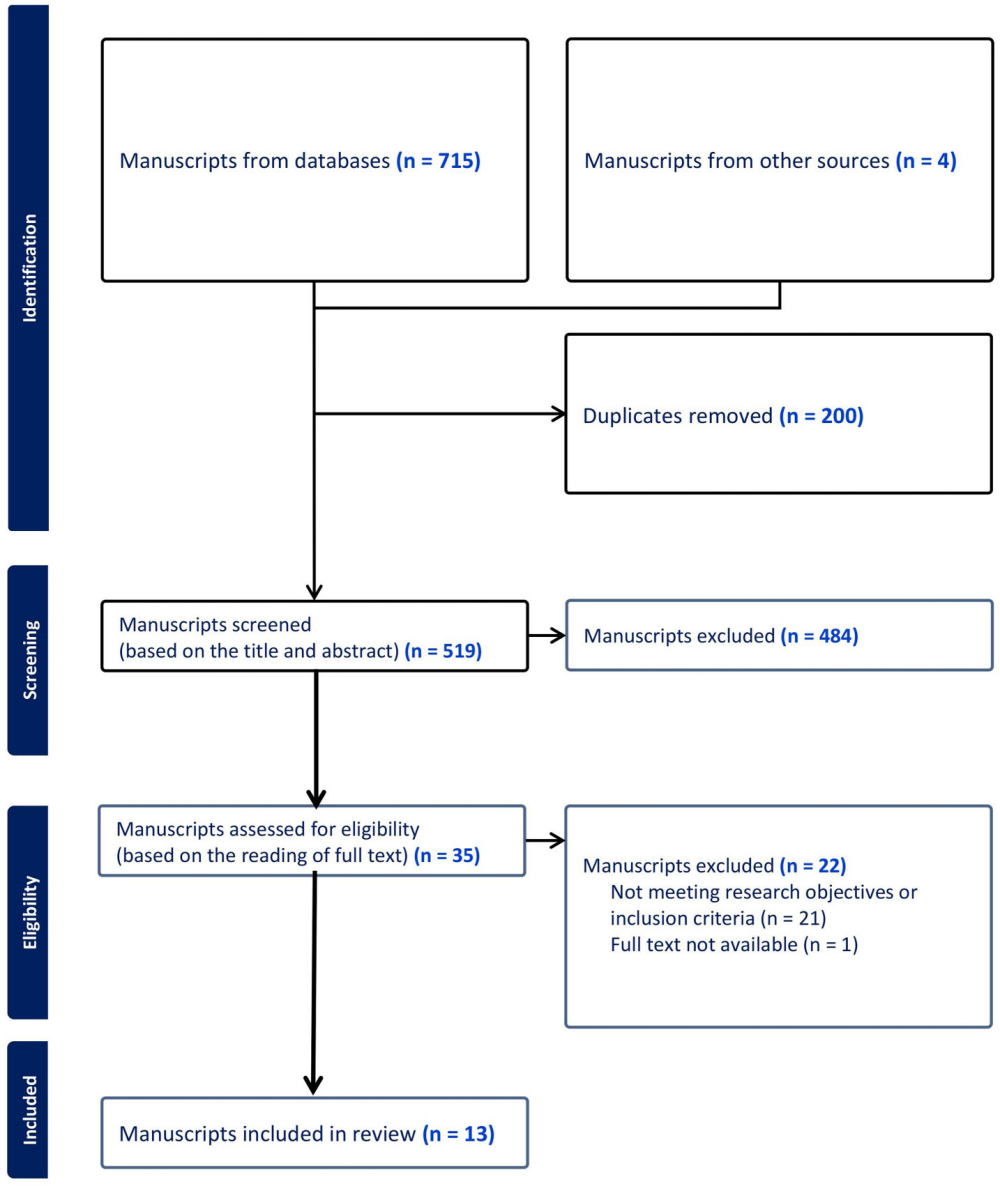
Manuscript eligibility determination process flowchart.

The manuscripts selected from the different databases were integrated into the reference management software Endnote and then into the web platform Covidence, which is dedicated to the management of literature reviews. Duplicates were eliminated, and the selection of manuscripts was performed in two steps based on the inclusion criteria outlined in Step 1.^40^ First, two evaluators individually selected from titles and abstracts, followed by selection from readings in full text. To include a manuscript in the study, both evaluators needed to agree on its relevance at each step. In case of disagreement, a third member of the research team was called upon to render the final decision. Regular meetings between evaluators took place to decide on the eligibility of manuscripts.^41^ This regular communication among evaluators strengthened their reflexivity and helped reduce the risk of personal bias.^42^ A PRISMA flowchart illustrates the flow of the manuscript selection process (see Figure 2).

#### Step 4: extracting the data

Two members of the research team independently reviewed and analyzed the literature presenting evaluation tools about the attributes of integrative prevention at work. To ensure a rigorous and systematic approach, they utilized an extraction grid that designed specifically for this study^32,43^ based on our chosen conceptualization of integrative prevention at work.^21^ This grid allowed the collection of information to answer the research objective (i.e., information about evaluation tools, such as their purposes, modality, and metrological properties), as well as descriptive details of the manuscripts (e.g., authors, publication date).^32^ For the first six manuscripts, two members of the research team carried out a pre-test of the extraction grid, each using the grid individually to extract data from the manuscripts. Subsequently, a debriefing meeting was held to put in common the extracted data and to modify and adjust the grid. This pre-test made it possible to verify that the grid allows the data to be extracted in a rigorous and constant manner so that the extracted information answers the research question optimally and transcends a mere summary of the manuscripts.^32,34^ The rest of the manuscripts were extracted by a single member of the research team, but regular meetings were held between members of the research team during this stage in order to ensure uniformity in the extraction process^42^

#### Step 5: collecting, summarizing, and reporting the results

The final step involved a two-part analysis. First, descriptive statistics (e.g., publication year, evaluation modality, assessment tools per attribute) were used to summarize the characteristics of the included manuscripts.^32,33,35,43,44^ Second, to address the research question, we used a descriptive approach to conduct a narrative synthesis of the findings.^42^ This synthesis involved systematically reviewing and thematically organizing the content of the included manuscripts. To facilitate this process, a table summarizing the major themes was developed.^42^ This table also served to highlight similarities and differences among the identified tools, as well as to identify gaps in the existing literature.^42^ This process was done by a single member of the research team and was verified periodically by another member.

## Results

### Description of the selected manuscripts

This scoping review led to the selection of 13 manuscripts that met the selection criteria. In those 13 manuscripts, 12 assessment tools were presented. Most of the manuscripts were published in the last 10 years (n = 8) and were written in English (n = 9). Half of the manuscripts were scientific articles (n = 7), while the other half were either reports (n = 5) or theses (n = 1). Most manuscripts addressed the attributes of communication among stakeholders (n = 10), collaboration among stakeholders (n = 10), and the holistic vision of health (n = 8), whereas the other attributes were less prevalent in the selected manuscripts. Table 1 presents the characteristics of the selected manuscripts.

**Table 1.** Characteristics of selected manuscripts (n = 13).

Number of manuscripts (%)	
*Publication date*	
2015 and after	8 (62%)
Before 2014	5 (38%)
*Publication language*	
English	9 (69%)
French	4 (31%)
*Country*	
Canada	6 (46%)
United-States	4 (31%)
India	1 (8%)
Multiple countries	1 (8%)
Unspecified	1 (8%)
*Document type*	
Scientific article	
Qualitative study	1 (8%)
Quantitative study	3 (23%)
Mixed methods	2 (15%)
Participatory research	1 (8%)
Report	5 (38%)
Thesis	1 (8%)
*Field of literature*	
Occupational therapy / rehabilitation	4 (31%)
Management	1 (8%)
Public health	4 (31%)
Organizational psychology	2 (15%)
Ergonomics	2 (15%)
*Evaluation modalities*	
Questionnaire	8 (62%)
Evaluation kit (contain observation grids, decision-support grids etc.)	3 (23%)
Evaluation guidelines	1 (8%)
Evaluative approach	1 (8%)
Attributes of integrative prevention at work addressed *	
Holistic vision of health	8 (62%)
Common understanding of the purpose of integrative prevention	1 (8%)
Communication among stakeholders	10 (77%)
Collaboration among stakeholders	10 (77%)
Coordination of preventive actions	4 (31%)

*Note: The total percentage exceeds 100% because some tools address more than one attribut.

### Narrative synthesis of tool assessing the attributes of integrative prevention at work

This synthesis presents the characteristics of 12 tools that assess one or more attributes of integrative prevention at work. Each tool is presented below in the same order: (a) its name, (b) its evaluation modality, (c) its purpose, (d) its position within the prevention continuum (health promotion, primary, secondary, tertiary prevention), (e) the attribute(s) it assesses, and (f) its metrological properties. Table 2 accompanies the narrative synthesis by summarizing the key findings.

**Table 2.** Summary of the characteristics of the assessment tools (n = 12).

Assessment tool name	Reference(s)	Assessment modality	Tool's purpose	Tool's place in the prevention continuum	Attribute assessed	Metrological properties
Return-to-work planning template (RTW-PT)	^44^	Questionnaire	Facilitate communication among stakeholders involved in a return-to-work process for cancer survivor.	Tertiary prevention	Holistic vision of health Communication among stakeholders Coordination of preventive actions	*Validity:* - Not reported *Reliability:* - Not reported
Health-and development-promoting leadership behavior questionnaire (HDLBQ)	^45^	Questionnaire	Assess leaders’ impact on workplace characteristics and workers’ well-being.	Primary prevention Secondary prevention Health promotion	Holistic vision of health Collaboration among stakeholders	*Validity* - Criterion validity (German version) was supported with workers’ well- being. - Factorial validity was verified *Reliability* - Excellent internal consistency levels - Excellent inter-items correlations
The Quality Criteria of Workplace Health Promotion	^46^	Questionnaire	Assess the quality of the health promotion actions in an organization.	Health promotion	Holistic vision of health Collaboration among stakeholders	*Validity*: - Not reported *Reliability*: - Not reported
The workplace integrated safety and health (WISH) assessment	^26,47^	Questionnaire	Assess an organization's best practice for health, safety, and well-being.	Primary prevention Secondary prevention Health promotion	Holistic vision of health Communication among stakeholders Collaboration among stakeholders Coordination of preventive actions	*Validity*: - Not reported *Reliability*: - Not reported
The questionnaire of the perceptions of safety officers	^48^	Questionnaire	Assess an organization's best practices for occupational health.	Primary prevention Secondary prevention	Communication among stakeholders Collaboration among stakeholders	*Validity:* - Factorial validity was verified. - Total variance explained by the questionnaire: acceptable (75%). *Reliability* - Internal consistency: high (Cronbach's alpha value was 0.817)
Psychosocial Factors Questionnaire 75 (PSF-Q75)	^49^	Questionnaire	Identify psychological risk factors in an organization, at different levels of analysis.	Primary prevention Secondary prevention	Communication among stakeholders Collaboration among stakeholders	*Validity* - Predictive validity with workers’ stress and motivation. - Construct validity is supported. *Reliability* - Internal consistency: Acceptable (Cronbach's alpha was between 0.66 to 0.70)
Mental Workload Questionnaire	^50^	Questionnaire	Assess the mental workload of a worker returning to work following a common mental disorder.	Tertiary prevention	Communication among stakeholders	*Validity*: - Content validity was verified with experts in health rehabilitation for common mental disorders in Quebec (Canada) *Reliability*: - Not reported
A guide to work constraints associated with musculoskeletal disorders	^51^	Evaluation kit	Assess risks related to musculoskeletal disorders to prevent occupational injuries or manage prolonged incapacity.	Primary prevention Secondary prevention Tertiary prevention	Holistic vision of health Communication among stakeholders Collaboration among stakeholders	*Validity*: - Not reported *Reliability*: - Not reported
Ergo Group	^52^	Evaluation kit	Assess risks work-situations for MSDs.	Primary prevention Secondary prevention	Communication among stakeholders Collaboration among stakeholders Coordination of preventive actions	*Validity*: - Not reported *Reliability*: - Not reported
Musculoskeletal Disorders – Guide and Tools for Work Retention and Return to Work	^53^	Evaluation kit	Identify work situations that need adaptations for workers with an MSD returning to work.	Tertiary prevention	Common understanding of the purpose of integrative prevention Communication among stakeholders Collaboration among stakeholders	*Validity*: - Not reported *Reliability*: - Not reported
The Creating a Way Forward Project	^54^	Evaluation guidelines	Assess the informational needs of workers living with chronic pain in a return-to-work process, as well as those of other stakeholders.	Tertiary prevention	Holistic vision of health Communication among stakeholders Collaboration among stakeholders	*Validity*: - Not reported *Reliability*: - Not reported
Intervention Design and Analysis Scorecard (IDEAS)	^30^	Evaluative approach	Engage frontline workers in the assessment and development of health promotion and preventive actions.	Primary prevention Secondary prevention Health promotion	Holistic vision of health	*Validity*: - Not reported *Reliability*: - Not reported

#### Return-to-Work planning template (RTW-PT)

The Return-to-Work Planning Template (RTW-PT) is a questionnaire available in two versions: one that can be completed by the worker and another that can be completed by their leader. Its aim is to improve communication among stakeholders involved with cancer survivors during their return to work.^45^ More specifically, this questionnaire serves as a means for the worker and the leader to assess their respective needs and expectations, to then be able to communicate them not only with each other, but also to other stakeholders who might be involved at the tertiary level of prevention (e.g., insurance case managers, primary care doctors). This questionnaire focuses on more than just occupational risks in the workplace; it assesses the resources, strengths, and challenges of the worker, the open-mindedness of the leader, and the like. Thus, it grants stakeholders a comprehensive understanding of the components influencing the worker's return to work, which is in line with a ***holistic vision of health***. Furthermore, the questionnaire contains a section dedicated to communication, which assesses the current state of the ***communication among stakeholders*** (e.g., whether there are times dedicated for communication) so that stakeholders can know whether measures are taken to facilitate the circulation of relevant information. This tool can help determine whether a variety of actions are integrated and whether stakeholders consider others’ actions during the return-to-work process so that beneficial effects are maximized and that the effects of the tertiary prevention actions are synergistic. Those aspects are crucial components of the ***coordination of preventive actions***. This tool has been the subject of a pilot study, but its metrological properties have not yet been evaluated.

#### Health- and development-promoting leadership behavior questionnaire (HDLBQ)

The Health- and Development-Promoting Leadership Behavior Questionnaire (HDLBQ) can be self-administered by either workers or leaders within an organization. This questionnaire aims to assess how leaders influence workplace characteristics and well-being, from their own perspectives as well as from workers’.^46^ Thus, this questionnaire focusses on health promotion, as well as primary and secondary prevention. The HDLBQ primarily focuses not only on leaders’ actions regarding working conditions, but it also assesses their impact on workers’ health and well-being, in terms of a ***holistic vision of health***. For instance, it allows organizations to assess whether leaders facilitate a good work–life balance for their workers. Some items of this questionnaire assess the ***collaboration among stakeholders***, particularly between leaders and their teams, by examining whether roles and responsibilities are understood and fulfilled effectively by both parties. This tool was first developed in Germany and is now validated in German, French, and English. The validation process involved various organizations from different sectors (e.g., information and technology, telecommunications) across Germany, France, and the United States. Factorial validity analysis confirmed that all items significantly contributed to their corresponding scales and that the factorial structure was equivalent across the three languages. The criterion validity of the German version of the HDLBQ with workers’ well-being was supported by the substantial relationships between all scales and indicators of workers’ well-being. Furthermore, these scales explained the incremental variance in workers’ well-being. Finally, in the samples for each country, all scales demonstrated adequate to excellent internal consistency levels and inter-item correlations.

#### The quality criteria of workplace health promotion

The Quality Criteria of Workplace Health Promotion offers a questionnaire that can be completed by any interested stakeholders (e.g., workers, leaders) in an organization. This questionnaire aims to help organizations assess the quality of their workplace health promotion programs by examining six key criteria.^47^ One of the six criteria emphasizes the integration of health promotion as a core responsibility within an organization's policies. The other five criteria concern workers’ involvement in the decision-making process, definition and planning of health promotion actions, corporate social responsibility, implementation of health-promoting measures, and evaluation of the consequences of health promotion. The criteria assessed by this questionnaire correspond to two of the attributes of integrative prevention at work. Some questions, such as “Do all staff have access to important health-related facilities (e.g., break and rest rooms, canteen, sports amenities)?” evaluate an organization's ***holistic vision of health*** by determining whether the organization promotes healthy lifestyles and encourages healthy behaviours at work, in addition to actions regarding occupational risks. Other questions assess the ***collaboration among stakeholders*** by examining the participation of stakeholders in health promotion actions (e.g., “Does everyone has chances to participate in decision making?”). Finally, no metrological properties were offered by the authors of this questionnaire.

#### The workplace integrated safety and health (WISH) assessment

The Workplace Integrated Safety and Health (WISH) assessment is a questionnaire that was developed for use by a team of stakeholders, ideally led by someone with in-depth knowledge of the organization's policies, programs, and practices that protect and promote workers’ health, safety, and well-being (e.g., human resource directors or executives in small businesses).^28,48^ The questionnaire aims to help stakeholders assess and critically examine the organization's actual best practices for primary and secondary prevention, as well as the promotion of workers’ health and well-being. The WISH assessment centers on six core constructs, each comprising targeted questions. One of them, *Policies, Programs, and Practices that Foster Supportive Working Conditions,* aligns closely with the attribute of a ***holistic vision of health,*** assessing whether the organization fosters healthy behaviours at work for workers by offering places where they can relax and take breaks, healthy food options, and similar amenities. It also assesses whether the organization makes efforts to prevent both mental and physical health issues (e.g., addresses both physical and psychological workplace violence). The constructs of *Leadership Commitment* and *Participation* include questions about ***communication among stakeholders***. They assess the communication status between leaders of various hierarchical levels and workers, examining whether workers are encouraged to participate in interactions and voice concerns regarding their health, safety, and well-being. ***Collaboration among stakeholders*** is assessed in this questionnaire through the *Participation* construct, which includes questions about workers’ inclusion in decision-making as well as preventive and health promotion actions. Similarly, the construct of *Comprehensive and Collaborative Strategies* briefly assesses the ***coordination of preventive actions*** by evaluating whether the health promotion and primary and secondary prevention actions are well-coordinated across all departments and stakeholders. The metrological properties of the WISH assessment have not yet been evaluated. However, the questions of the tool are based on high internal consistency indicators that demonstrated convergent validity with occupational safety and health, as well as health promotion activities and policies. The metrological properties of this tool are expected to undergo an evaluation process in the future.

#### The questionnaire of the perceptions of safety officers

The Questionnaire of the Perceptions of Safety Officers was developed by Shankar et al.^49^ This questionnaire aims to help individuals responsible for health and safety in the workplace assess occupational health and safety practices in their organization, specifically at the primary and secondary prevention levels, based on their perceptions. This questionnaire consists of 23 items divided into nine scales. Some items in this questionnaire directly assess the attributes of integrative prevention at work. For example, the item “A format on functions of commitment, participation, and responsibilities is established on all safety aspects and available to all organization members” reflects the attribute of ***the collaboration among stakeholders***, emphasizing the importance of everyone understanding their roles and the roles of others, which is a crucial component for their engagement with one another. Other items also assess the ***communication among stakeholders*** by assessing whether information circulates effectively among everyone and whether opportunities for dialogue are provided to everyone concerned with the preventive actions. The questionnaire was validated in the construction, steel, and refractory industries in India. A factor analysis was conducted, and only items with factor loadings above 0.6 were retained in the final version. The total variance explained by the questionnaire is acceptable (75%). Additionally, the overall internal consistency, measured using Cronbach's alpha, was 0.817, which is above the acceptable value.

#### Psychosocial factors questionnaire 75 (PSF-Q75)

The Psychosocial Factors Questionnaire 75 (PSF-Q75) is a questionnaire intended for use by various stakeholders of an organization, such as workers or leaders, except for higher-level management.^50^ This self-administered questionnaire aims to help its users identify psychosocial risk factors that may or may not be present in their workplace at different analysis levels (i.e., job, group, and organization). The questionnaire takes a holistic approach to assess the psychosocial risks that workers may potentially or currently face within the context of managing working conditions, aligning with the primary and secondary levels of prevention. The PSF-Q75 assesses two attributes of integrative prevention at work: communication and collaboration. ***Communication among stakeholders*** is assessed through various items, such as “In my workgroup, communication is good” or “In my workgroup, information sharing is good,” which clearly allows the assessment of the quality of communication and information sharing among stakeholders. ***Collaboration among stakeholders*** is also evaluated with items like “In my workgroup, there are opportunities to participate in decision-making,” emphasizing the importance of equal participation from all stakeholders in decision-making for preventive actions. This questionnaire was validated with a Latin American population at a large organization in the food sector. A confirmatory factor analysis demonstrated an acceptable goodness-of-fit for its structure. Evidence was found for the predictive validity of the items on workers’ stress and motivation. Convergent and discriminant validity were overall supported for all factors in this questionnaire. Lastly, all the scales demonstrated acceptable internal consistency.

#### Mental workload questionnaire

The Mental Workload Questionnaire [*Questionnaire de la charge de travail mentale*] (QCTM), developed by Tremblay-Boudreault,^51^ is a questionnaire designed to support health professionals in rehabilitation (e.g., occupational therapists) and workers in their return-to-work process following an absence due to common mental disorders. This questionnaire aims to assess the mental workload (both underload and overload) experienced by the worker, which can pose an obstacle to both returning to and/or staying at work. Workers can complete the questionnaire on their own, providing rehabilitation professionals with insights to better understand workers’ challenges and adapt their therapeutic intervention (i.e., actions at the tertiary level of prevention) accordingly. Although the primary focus of this questionnaire is to assess a worker's mental workload to implement actions aligned with the worker's challenges, ***communication among stakeholders*** is also evaluated. In fact, this questionnaire can provide an overview of the communication status among stakeholders. It can help detect inconsistencies in what other stakeholders (e.g., leader, primary care doctor) tell the worker, thereby ensuring that the correct information circulates effectively. The questionnaire's content validity was verified by experts in health rehabilitation for common mental disorders (e.g., occupational therapists, psychologists) in Quebec, Canada, but no other metrological properties were presented.

#### A guide to work constraints associated with musculoskeletal disorders

A Guide to Work Constraints Associated with Musculoskeletal Disorders is offered by Stock et al.^52^ This evaluation kit outlines steps for assessing occupational risks related to musculoskeletal disorders that workers could or currently face, with the aim of preventing occupational injuries and managing incapacity. The guide, acting in primary, secondary, and tertiary prevention, also suggests various complementary assessment modalities (e.g., questionnaires, observation grids). The choice of complementary assessment modalities is left to the discretion of the stakeholder using the guide because it is intended mainly for professionals with training in ergonomics or related fields (e.g., occupational therapists), allowing them to make clinical judgments in these situations. However, the ergonomist (or other professional) should perform the evaluation together with other stakeholders from the organization (e.g., workers and leaders). This guide allows professionals and stakeholders to conduct a comprehensive assessment of occupational risks that could lead to musculoskeletal disorders in workers, which is in line with a ***holistic vision of health.*** In fact, it allows stakeholders to assess not only occupational risks associated with the physical dimension of workers (e.g., restrictive positions), but also risks linked to psychosocial (e.g., violence at work) or organizational dimensions (e.g., working conditions), thereby considering the work situation in its entirety. This guide helps to assess ***communication among stakeholders*** by allowing them to evaluate whether they believe that information circulates about ongoing or upcoming prevention initiatives. Furthermore, ***collaboration among stakeholders*** can be assessed through this guide, which helps critically examine whether every relevant stakeholder can participate in preventive actions and decision-making. No metrological properties were presented.

#### Ergo group

The “Ergo Group” [*Groupes ergo*] evaluation kit, developed by St-Vincent et al.,^53^ uses evaluation grids, observation tools, and interview guides to support users—which should be a multidisciplinary work committee with stakeholders of varied expertise (e.g., ergonomists, workers, engineers). This evaluation guide was designed to help such a committee assess and improve working conditions within an organization related to pain associated with musculoskeletal disorders (MSDs), using a participatory ergonomics approach.^53^ The committee assesses high-risk work situations and then proposes and assesses primary and secondary prevention measures. This guide provides a clear understanding of the ***coordination of preventive actions*** within an organization. It allows the committee to observe whether diverse actions are being implemented and coordinated to prevent MSDs at each level of prevention (i.e., primary and secondary levels in this approach). Being team-based, the Ergo group involves ***communication among stakeholders*** and ***collaboration among them*** but does not assess these attributes directly. However, the success of the group relies on regular reflections by members to ensure that these two attributes are present. No metrological properties were discussed by the authors.

#### Musculoskeletal disorders – guide and tools for work retention and return to work

Musculoskeletal Disorders – Guide and Tools for Work Retention and Return to Work [*Troubles musculo-squelettiques - Guide et outils pour le maintien et le retour au travail*] by Stock et al.^54^ is an evaluation kit containing four series of forms and decision support grids intended for stakeholders of an organization (e.g., leaders, human resources directors) and others offsite (e.g., primary care doctors, physical therapists) who are involved in return-to-work processes. This evaluative guide aims to assess the elements needed to adapt the return-to-work process following a musculoskeletal disorder, thereby minimizing the risk of worsening workers’ physical injuries. Thus, this evaluative guide supports stakeholders doing tertiary prevention. It also allows stakeholders to focus more closely on the issues and needs of injured workers and facilitates their communication. Moreover, this guide can help assess some dimensions of the ***communication among stakeholders***. It helps stakeholders assess whether they know who needs to communicate with whom in different scenarios (e.g., who should inform the primary care doctor if a worker faces challenges during their return to work) and what communication methods should be used (e.g., phone calls, written reports), so that information circulates fluidly among everyone. Furthermore, the guide helps stakeholders assess whether the worker can participate on equally terms with other stakeholders in their return-to-work process (e.g., whether they can give their opinion concerning some stakeholders’ actions and be heard), which aligns with the ***collaboration among stakeholders***. This guide also helps stakeholders to determine whether they all have a ***common understanding of the purpose of integrative prevention*** (i.e., what they want to achieve, their deadlines); if not, steps are offered to guide them to find one and work towards it. No metrological properties were documented for this guide.

#### The creating a way forward project

The Creating a Way Forward Project is a set of evaluation guidelines that aim to identify the informational needs of workers living with chronic pain, as well as of the stakeholders involved with them (e.g., leaders, health professionals, insurers), relating to the subject of returning or staying at work while living with a chronic pain condition.^55^ To identify these needs, the workers and stakeholders involved can use the evaluation guidelines, which are divided into five categories that represent their potential informational needs. These guidelines help workers identify what they would need to know in order to return to, or remain at work in a healthy way, and the stakeholders what information they need to optimally conduct tertiary prevention actions. Of these five categories, one focuses on holistic approaches to chronic pain treatment and management; it is linked to a ***holistic vision of health***. Questions are posed to the workers, so they can assess whether they have or need strategies to self-manage their condition at work. These reflection elements guide workers to go beyond strategies for merely avoiding occupational risks by helping them develop strategies to engage in healthy behaviours at work that help them maintain their health and well-being. Stakeholders are offered similar reflection elements so that they can question themselves as to how they can foster this holistic approach to health. Another category emphasizes assessing ***collaboration among stakeholders*** by prompting workers and stakeholders to question themselves about their respective roles and responsibilities, as well as what the roles of other stakeholders in the return or stay-at-work process are (i.e., tertiary prevention). A third category offers reflection questions on communication, which allow users to evaluate the ***communication among stakeholders.*** In fact, it can help workers and stakeholders assess whether they know to whom they should communicate in different scenarios (e.g., whom the workers can contact if they need moral support), so that information circulates effectively, and the workers can signal their needs and issues to the right people. No metrological properties were documented for this tool.

#### The intervention design and analysis scorecard (IDEAS) tool

The Intervention Design and Analysis Scorecard (IDEAS) tool is an evaluative approach divided into seven steps. It aims to engage frontline workers in the development and assessment of health promotion actions as well as primary and secondary preventive actions.^31^ This evaluative approach offers scorecards to assess different health promotion and preventive actions that workers would like to see implemented by their organizations (e.g., organize the workflow to allow more rest for reducing back pain). The scorecards help workers assess the relevance of a chosen action in terms of its costs, resources needed, potential benefits or barriers. Thus, this tool offers a structured evaluative approach for workers to select actions that are aligned with their organizational context. The IDEAS tool facilitates the assessment of preventive actions and health promotion actions, which is in the spirit of a ***holistic vision of health***. The IDEAS tool integrates not only the assessment of primary and secondary actions but also health promotion actions. Furthermore, the structure offered by the scorecard and its way of considering a set of factors beyond occupational risks (e.g., resources needed, costs) help workers assess actions that affect their physical and mental health, as well as their well-being. This tool currently has no documented metrological properties.

### Comparative analysis of the assessment tools

A comparative analysis of the 12 tools reveals variations in their scope across the prevention continuum, the number and combination of attributes assessed, their metrological robustness, and the degree of professional expertise required for their implementation.

While several tools address multiple levels of prevention, such as the WISH assessment,^28,48^ HDLBQ,^46^ and A Guide to Work Constraints Associated with Musculoskeletal Disorders,^52^ others focus more narrowly on a single level, such as tertiary prevention in the case of the RTW-PT,^45^ the Mental Workload Questionnaire,^51^ The Creating a Way Forward Project,^55^ and Musculoskeletal Disorders – Guide and Tools for Work Retention and Return to Work.^54^ Furthermore, the number and combination of attributes assessed vary notably. Some tools assess three (e.g., Ergo Group^53^), or even four attributes (e.g., WISH assessment^28,48^) of integrative prevention at work, suggesting a broader coverage of the concept, whereas others focus more specifically on one (e.g., IDEAS tool^31^) or two attributes (e.g., The Quality Criteria of Workplace Health Promotion^47^). It is also noteworthy that although many tools target health promotion, few (e.g., HDLBQ,^46^ IDEAS tool^31^) explicitly incorporate it alongside primary and secondary prevention. In terms of metrological robustness, only a minority of tools (i.e., HDLBQ,^46^ PSF-Q75,^50^ and Perceptions of Safety Officers^49^) report psychometric validation, while many others lack documented evidence of validity or reliability. While most tools are self-administered and designed for internal use within organizations, one tool (i.e., the Guide to Work Constraints Associated with Musculoskeletal Disorders^52^) relies on a structured process led by external experts, potentially limiting accessibility for organizations but enhancing assessment accuracy and depth.

## Discussion

This study described a number of evaluation tools used to assess attributes of integrative prevention at work. Following a scoping review design, analysis of 13 manuscripts led to the identification of 12 evaluation tools that were described according to different information (e.g., their purpose, the attribute(s) they assess, their metrological properties). The results of this study contribute to the advancement of knowledge based on three main ideas: (1) the importance of the empowerment of stakeholders through self-administered tools, (2) the importance of assessing communication and collaboration, and (3) the potential to assess the common understanding of the purpose of integrative prevention. Avenues for future research are also proposed.

### Empowering stakeholders through self-administered tools for integrative prevention at work

This study proposed 12 assessment tools that evaluate one or more attributes of integrative prevention at work. Among these tools, nearly all (11) were designed to be self-administered, highlighting the importance of stakeholders’ empowerment in assessing the presence of the attributes of integrative prevention at work within organizations and improving practices accordingly.^28,56^ In other words, evaluations benefit from being conducted, or at least facilitated, by those who implement prevention practices.^57^ Increasingly, the development of simple tools that can be used by stakeholders involved in prevention initiatives within their own environments is being presented as a solution to prevention challenges in the workplace (e.g., for assessing the implementation of return-to-work interventions).^58^ The self-administered approach is advantageous also in that the user completing the assessment tool is well acquainted with the organizational context of the evaluation.^59^ Generally, self-administered tools are valued for their ease of use (e.g., requiring minimal time and financial resources)^60^ and for allowing respondents to develop critical thinking about what they are evaluating.^61^ However, self-administered tools may yield responses that diverge from actual facts.^62^ For complex issues, it may be useful to forgo this type of administration and involve an external expert (e.g., an ergonomist) to evaluate the situation. Experts can provide an accurate interpretation of the assessed data, offering a clear and comprehensive view of reality.^63^ Furthermore, we recommend that self-administered tools be used in combination with other sources of information, such as complementary tools or organizational indicators, to ensure a more robust and accurate assessment of workplace prevention practices. This triangulation approach can help mitigate potential biases associated with self-reporting and reduce the risk of drawing incomplete or misleading conclusions in practice. Nevertheless, the results of this study highlight the importance of a self-administered tool that enables organizational stakeholders to make relevant changes on their own. This finding complements other studies showing the benefits of using self-administered tools in the workplace.^64,65^ For example, Descatha et al.^64^ found that self-administered questionnaires were more accurate than direct third-party observations in identifying the risk exposure of workers performing repetitive tasks. Furthermore, numerous studies in healthcare have shown that the use of self-administered tools fosters user empowerment and autonomy.^66–68^ For example, Downey et al.^67^ found that patients in emergency settings who discovered post-traumatic stress disorder through a self-administered tool were significantly more likely to seek follow-up care compared to those whose questionnaires were administered by a third party. Our study suggests that such benefits could be observed for organizational stakeholders as well. In short, a self-administered assessment tool appears to be the ideal approach for performing assessments in the context of occupational injury and prolonged incapacity at work.

### The importance of assessing communication and collaboration among stakeholders

The findings of this study highlight two attributes of integrative prevention at work that are frequently assessed in the context of preventing occupational injuries and managing incapacity at work. Communication among stakeholders and collaboration among stakeholders were evaluated by more than three-quarters (77%) of the tools, which indicates the importance of assessing these two attributes in the context of occupational injuries and prolonged incapacity at work. Such assessments help enhance understanding of what contributes to the success or failure of prevention initiatives. Communication and collaboration among stakeholders is also evaluated in other contexts to better understand complex situations. For example, in civil engineering, communication among the various parties involved in a construction project is used as an indicator to understand and address performance issues (e.g., a lack of communication among on-site workers and the client causes delays and increases construction costs).^69^ Similarly, the evaluation of communication serves as a lever for improving patient care; it is assessed among resident physicians through self-administered questionnaires to help make them aware of their interactions with patients and improve their practice.^70^ Similar to communication, the evaluation of collaboration has been proposed as an effective way to better understand and address various issues, such as violence in schools.^71^ In short, the assessment of communication among stakeholders as well as collaboration among them could be a valuable practice in the context of preventing and managing occupational injury and prolonged incapacity at work.

### Common understanding of the purpose: an overlooked attribute in the evaluation of integrative prevention at work

Few of the tools described in this study evaluate common understanding of the purpose of integrative prevention. Although many manuscripts alluded to the importance of stakeholders having a shared goal (e.g.,^28^) or offered strategies for establishing one (e.g.,^31^), only one of the tools offered a concrete means of assessing the presence of a common understanding of the purpose of integrative prevention.^54^ Indeed, few evaluation tools in the field of work emphasize and concretely assess this attribute. For example, to foster organizational motivation in the context of sales and customer service, Gershfeld^72^ proposed that stakeholders could assess, through various questions in a questionnaire she proposes, whether they have a common understanding of the goal to be achieved. Among other things, the questions evaluate whether the goal is well identified and known by all; Gershfled's questionnaire even goes so far as to assess and help plan actions to be implemented to achieve the goal. In this case, assessing the common goal is identified as an essential lever for mobilizing the organization into action. Several authors (e.g.,^73^) have emphasized the importance of identifying and having a common goal in organizational contexts, but few go further to evaluate it using concrete means, even though assessing such an attribute could promote its operationalization in the workplace.^20^ In summary, our study suggests that formally assessing the common understanding of the goal may be relevant to the prevention of occupational injuries and the management of incapacity at work.

### Strengths and limitations

The methodology chosen for this study allowed us to obtain what is, to our knowledge, the first comprehensive and detailed overview of currently available tools for assessing the attributes of integrative prevention at work. This overview presents evaluation tools based on the most recent conceptualizations of integrative prevention at work. The use of a systematic, well-defined, step-by-step approach—along with the methodological details provided in this article—ensures reproducibility and attests to the rigor of this study. However, the methodology employed is limited in that the quality of the included manuscripts was not assessed. Hence, while we provided a comprehensive overview of existing evaluation tools, we did not assess the robustness of the evidence supporting each tool. Although the overview of evaluation modalities is up to date, it reflects the assessment of an emergent concept that is continuously evolving. It is therefore possible that this overview may require updating in the future.

### Future research avenues

The results of this study highlight several gaps in the tools used to assess the attributes of integrative prevention at work. First, analysis revealed the absence of any assessment tool capable of assessing all five attributes of integrative prevention at work on a unified scale, although some tools could assess up to three^45,52–55^ or even four^28,48^ attributes. The results also emphasize gaps in the metrological properties of the available tools. Indeed, only four tools have been evaluated for their metrological properties.^46,49–51^ Furthermore, some authors pointed out shortcomings in the validation of these tools, such as preliminary evaluations that would require further investigation (e.g., the QCTM has been assessed only for content validity).^51^ Moreover, these tools are validated in specific organizational contexts, which may limit their applicability to other settings. Finally, several of the available evaluation tools were developed more than a decade ago (e.g.,^47^), potentially rendering them obsolescent or less apt to reflect the contemporary conceptualization of integrative prevention at work. To address these gaps, this study underscores the potential need to create and validate a new, unified evaluation tool that encompasses all attributes of integrative prevention at work, thereby enabling organizations to effectively implement this innovative approach to prevention. While the development of a unified evaluation tool is needed, we suggest that organizations can, in the meantime, benefit from combining existing tools that target complementary attributes to support the actualization of integrative prevention at work. Future research could focus on appraising the quality of the evidence supporting each evaluation tool to better understand the robustness of their validation processes and their applicability across diverse organizational contexts.

## Conclusion

Integrative prevention at work is a promising avenue for reducing occupational injuries and prolonged incapacity in the contemporary world of work. To operationalize this concept in organizations, it was necessary to evaluate its five attributes. Therefore, using a scoping review design, this study described 12 evaluation tools that assess one or more attributes of integrative prevention at work. Its results highlight the importance of the self-evaluation of prevention actions by stakeholders, as well as the importance of assessing each attribute, including communication and collaboration among stakeholders as well as common understanding of the purpose of the preventive actions. The results show that no current tool assesses all five attributes on the same scale. To enable organizations to benefit from integrative prevention at work, the logical next step would be to develop and validate a new assessment tool that can comprehensively evaluate integrative prevention at work.

## Supplemental Material

sj-docx-1-wor-10.1177_10519815251383525 - Supplemental material for Assessing integrative prevention at work: A scoping review Supplemental material, sj-docx-1-wor-10.1177_10519815251383525 for Assessing integrative prevention at work: A scoping review by Andrée-Anne Drolet, Alexandra Lecours and Lily Bellehumeur-Béchamp in WORK

sj-docx-2-wor-10.1177_10519815251383525 - Supplemental material for Assessing integrative prevention at work: A scoping review Supplemental material, sj-docx-2-wor-10.1177_10519815251383525 for Assessing integrative prevention at work: A scoping review by Andrée-Anne Drolet, Alexandra Lecours and Lily Bellehumeur-Béchamp in WORK
