# Supplementary material for: Assessing integrative prevention at work: A scoping review
Source: Work. 2025 Nov 4;83(4):931–47. doi: 10.1177/10519815251383525 (PMC13053870; doi:10.1177/10519815251383525)
Supplement: sj-docx-1-wor-10.1177_10519815251383525 - Supplemental material for Assessing integrative prevention at work: A scoping review [file sj-docx-1-wor-10.1177_10519815251383525.docx]

**Appendix 1.** Example of search strategy used.

| **Search strategy** | **Database** |
| --- | --- |
| TI ( “Integrat* prevention” OR “integrat* management” OR “integrat* approach*” OR “comprehensive approach*” OR "holistic approach*" OR "integrat* intervention*" OR "holistic vision" OR (shared OR common) W3 (goal OR purpose) OR (communication N1 stakeholders) OR (collaboration N1 stakeholders) OR (coordination N2 action*) ) OR AB ( “Integrat* prevention” OR “integrat* management” OR “integrat* approach*” OR “comprehensive approach*” OR "holistic approach*" OR "holistic vision" OR (shared OR common) W3 (goal OR purpose) OR (communication N1 stakeholders) OR (collaboration N1 stakeholders) OR (coordination N2 action*) )  AND  TI ( workplace* OR "work location*" OR "work site*" OR "work place*" OR "job site*" OR "worksite*" OR “work environment” ) OR AB ( workplace* OR "work location*" OR "work site*" OR "work place*" OR "job site*" OR "worksite*" OR “work environment” ) OR DE "Workplace Intervention"  AND  TI ( “assessment tool*” OR “assessment instrument*” OR “evaluation tool*” OR “evaluation instrument*” OR “indicator tool*” OR “measurement tool*” OR “quality checklist*” OR “quality scale*” OR “quality tool*” OR “screening tool*” OR “self-assessment tool*” OR “tool*” OR “tool* for assessing” OR “tool* for evaluating” OR “tool* for measuring” OR “tool* for screening” OR “tool* to assess” OR “tool* to evaluate" OR “questionnaire*” OR empirical OR guide* OR checklist* ) OR AB ( “assessment tool*” O [...](javascript:showHistoryTerm('ctl00_ctl00_FindField_FindField_historyControl_HistoryRepeater_ctl00_ellipsis',true)) OR DE "Questionnaires" OR DE "Evaluation" OR DE "Measurement" OR DE "Testing" | PsycInfo |
