# Supplementary material for: Assessing integrative prevention at work: A scoping review
Source: Work. 2025 Nov 4;83(4):931–47. doi: 10.1177/10519815251383525 (PMC13053870; doi:10.1177/10519815251383525)
Supplement: sj-docx-2-wor-10.1177_10519815251383525 - Supplemental material for Assessing integrative prevention at work: A scoping review [file sj-docx-2-wor-10.1177_10519815251383525.docx]

**Appendix 2**. PRISMA -ScR Checklist.

| **SECTION** | **ITEM** | **REPORTED ON PAGE #** |
| --- | --- | --- |
| Title | 1 | P.1 |
| Structured summary | 2 | P.2 |
| Rationale | 3 | P.3-6 |
| Objectives | 4 | P.6 |
| Protocol and registration | 5 | *A review protocol does not exist* |
| Eligibility criteria | 6 | P.7 |
| Information sources | 7 | P.7-8 |
| Search | 8 | Appendix 1 |
| Selection of sources of evidence | 9 | P.8 |
| Data charting process | 10 | P.8-9 |
| Data items | 11 | *Not applicable* |
| Critical appraisal of individual sources of evidence | 12 | *No critical appraisal was done.* |
| Synthesis of results | 13 | P.9 |
| Selection of sources of evidence | 14 | P.9 ; table 1 |
| Characteristics of sources of evidence | 15 | P.10 ; table 1 |
| Critical appraisal within sources of evidence | 16 | *Not applicable* |
| Results of individual sources of evidence | 17 | P.10-18 |
| Synthesis of results | 18 | Table 2 |
| Summary of evidence | 19 | P.18-19 |
| Limitations | 20 | P.21-22 |
| Conclusions | 21 | P.23 |
| Funding | 22 | P.24 |
